# Development and validation of an interpretable machine learning model for predicting the risk of 8-year all-cause mortality in Cardiovascular-Kidney-Metabolic Syndrome among older adults: A multicenter and cohort study

**DOI:** 10.21203/rs.3.rs-8473902/v1

**Published:** 2026-01-25

**Authors:** Zhiren Zhu, Jie Zhang, Peirao Wu, Huiping Xue, Dongmei Gu

**Affiliations:** Affiliated Hospital of Nantong University; Nantong University; Nantong University; Affiliated Hospital of Nantong University; Affiliated Hospital of Nantong University

**Keywords:** Cardiovascular-kidney-metabolic syndrome, 8-year all-cause mortality, Machine learning, Interpretable, Predictive model, Multicenter and cohort study

## Abstract

**Background:**

Cardiovascular-kidney-metabolic syndrome (CKM) among elder adults due to age-related physiology is a high burden, but long-term mortality risk prediction is understudied. This study aims to develop and validate an explainable machine learning model to predict 8-year all-cause mortality.

**Methods:**

This study used HRS (2012–2020) and CHARLS (2011–2020) database, performed data cleaning and multiple imputation, plotted Kaplan–Meier curves with log-rank tests, conducted competing risk analyses, and pooled estimates using Rubin’s rules. Cox regression was used to adjust for confounders. In CHARLS, variables were selected using LASSO (retained if selected ≥ 70%), after sensitivity and collinearity checks. We compared six survival models with 10-fold cross-validation and evaluated performance with AUC, DCA, calibration curves, and the Brier score, with external validation in HRS. SHAP was used to explain feature importance.

**Results:**

8,473 participants (CHARLS 4,460; HRS 4,013) was included. Pooled 8-year mortality by CKM stages 0–4 was: HRS 9.2%, 4.94%, 9.14%, 12.57% and 18.97%; CHARLS 15.69%, 9.92%, 16.76%, 25.51% and 26.19%. Each one-stage increase in CKM was associated with a 40% and 15% higher mortality risk (both P < 0.001). The Cox model performed best: internal AUC 76.7% (95%CI: 76.5%–76.8%); external AUC 76.6% (95%CI: 76.2–77.0). Top three SHAP features: age, smoking and cystatin C.

**Conclusions:**

The Cox model showed good discrimination, calibration, and interpretability for predicting 8-year mortality risk in older adults with CKM syndrome across Chinese and American populations. Research findings indicate that interventions targeting the predictors cystatin C and gait speed may help reduce the long-term risk of mortality.

## Introduction

Noncommunicable diseases (NCDs) pose a global public health challenge, causing approximately 41 million deaths annually, accounting for about 74% of all deaths worldwide [1]. Cardiovascular disease (CVD), kidney disease, and metabolic disorders (such as type 2 diabetes) frequently coexist, are pathophysiologically interconnected, and significantly increase mortality risk [2–4]. Recognizing these interactions, the American Heart Association (AHA) Presidential Advisory proposed the Cardiovascular-Kidney-Metabolic (CKM) Syndrome, which stratifies individuals into five progressive stages on the basis of metabolic risk, kidney function, and CVD risk to strengthen multidisciplinary approaches to prevention, risk stratification, and management [5].

Consistent with this framework, the 2021 Global Burden of Disease (GBD) study reports that, among adults aged ≥ 55 years, CVD remained the leading cause of death from noncommunicable diseases from 2017 to 2021, with a consistent upward trend. In 2021, there were 17,348,107 CVD deaths, led by ischemic heart disease and stroke as the top two CVD causes. Mortality from diabetes and chronic kidney disease likewise increased annually, totaling 2,724,913 deaths in 2021 and ranking fourth overall; diabetes and CKD were the fifth and sixth leading individual causes of death, respectively [6]. Poor CKM health is a major determinant of all-cause mortality, posing a significant burden on global public health systems and socio-economics [7].

Although epidemiological studies on CKM syndrome remain limited, existing studies consistently indicate a high population burden with meaningful heterogeneity and prognostic implications. Specifically, analyses based on the U.S. National Health and Nutrition Examination Survey (NHANES) database indicate that CKM syndrome is prevalent among U.S. adults, with its prevalence increasing with age and varying across different genders, racial and ethnic groups [8, 9]. Analyses based on the China Health and Retirement Longitudinal Study (CHARLS) indicate that CKM syndrome is also prevalent among Chinese adults, with prevalence patterns similar to those observed in the United States [10]. In a large prospective cohort study from the UK, 80% of participants were classified as CKM stages 2–4, and CKM stage was positively associated with all-cause mortality, with risk increasing across stages; by stage 4, the all-cause mortality rate reached 30.09%. Using stage 0 as the reference and adjusting for variables, the hazard ratio (HR) for all-cause mortality at stage 4 was 2.13 (95% CI, 1.94–2.34) [11].

Adults ≥ 60 years are more likely to present with advanced CKM stages, and CKM prevalence rises with age [11]. Most studies evaluate mixed-age populations, with insufficient focus on older adults, who bear the highest absolute mortality risk and often have multimorbidity. CKM stages for long-term outcomes such as 8-year all-cause mortality are scarce, and few studies rigorously assess external validity across countries. Critically, current risk stratifications mainly rely on broad staging systems, potentially overlooking complex, non-linear interactions among risk factors in older adults, and few studies have rigorously assessed the external validity of predictive models across different countries.

Machine learning (ML) has recently demonstrated exceptional performance in clinical prognosis and diagnostic prediction due to its powerful ability to handle complex interactions and nonlinear relationships, and it is being increasingly applied in clinical research [12–15]. Addressing the black boxes issue, the introduction of model interpretability technology is crucial. By revealing the decision-making process of algorithms, it enhances model transparency, helping clinicians understand and trust model outputs, thereby accelerating the practical application of artificial intelligence in clinical diagnosis and treatment [16].

To address these gaps, this study aimed to develop and validate an interpretable machine learning (ML) model using longitudinal data from CHARLS and Health and Retirement Study (HRS) to predict the risk of 8-year all-cause mortality among older adults with CKM syndrome and to assess the feasibility of explainable ML models in clinical practice, with the goal of informing clinical decision-making and interventions.

## Methods

### Study design

This is a multicenter and cohort study. We generated Kaplan–Meier (KM) survival curves for all patients and conducted significance testing. To predict the 8-year mortality risk among patients with CKM syndrome from the diagnostic baseline, we developed and compared six modeling approaches: two regression methods (Cox proportional hazards and competing risks regression) and four machine-learning methods (Random Survival Forests, Survival Gradient Boosting, XGBoost survival, and Survival Decision Tree). To ensure transparent and complete reporting of AI-based prediction model development and evaluation, we adhered to TRIPOD (Transparent Reporting of a multivariate predictive model for Individual Prognosis or Diagnosis) [17].

### Sample size calculations

The sample size for this study was determined using the events-per-variable (EPV) methods [18, 19]. According to the UK Biobank data, CKM stages (0-4) exhibited all-cause mortality rates ranging from 6.32% to 30.1% over 14.7 years of follow-up period [11]. The study population comprised CKM patients aged ≥60 years, predominantly in stages 3–4, so the study selected 30.1% as the projected 8-year all-cause mortality rate and planned including 10 candidate predictor variables and sets the EPV to 20. We calculated the required sample size using the following formula:

$$\text{Sample Size =}\frac{\text{Number of Variables×EPV}}{\text{Incident Rate}}=\frac{10\times 20}{0.301}\approx 665$$

Considering a 15% non-response rate, we ultimately require 783 participants.

### CKM syndrome stages and outcome definitions

CKM syndrome followed the 2023 American Heart Association (AHA) Presidential Advisory was defined from 0 to 4 [5]. Stage 0 (No risk factors): individuals with normal body mass index (BMI) and waist circumference, normal glycemia, blood pressure, and lipids, and no metabolic risk factors (diabetes, hypertension, hypertriglyceridemia), with no evidence of CKD or subclinical/clinical CVD; Stage 1 (Excess or dysfunctional adiposity): population with overweight/obesity, abdominal obesity, or adipose tissue dysfunction, without other metabolic risk factors or CKD; Stage 2 (Metabolic risk factors and CKD): metabolic risk factors or moderate-to high-risk CKD, or both; Stage 3 (Subclinical CVD in CKM): meeting Kidney Disease: Improving Global Outcomes (KDIGO) criteria for very high-risk CKD or with a predicted 10-year CVD risk ≥20%; Stage 4 (Clinical CVD in CKM): clinical CVD among individuals with excess/dysfunctional adiposity, other CKM risk factors, or CKD.

The primary outcome of this study was all-cause mortality from baseline to 8 years. Participants with predetermined mortality risks, such as those with terminal cancer, were excluded at baseline. Deaths were recorded with exact dates; when only vital status was known without a date, the midpoint between adjacent survey waves was imputed. Then we conducted statistical modeling by outcome status, generated KM curves, and developed ML models to predict mortality risk.

### Data sources and study population

The study is based on two nationwide longitudinal cohort studies: CHARLS and HRS. CHARLS covers 150 counties in 28 provinces and began in 2011 with over 17,000 enrollees, targeting policy and health issues among Chinese adults aged ≥45 years [20]. All participants completed standardized questionnaires to collect sociodemographic, lifestyle, and health-related data. HRS has followed a nationally representative sample of U.S. adults aged ≥50 years every two years since 1992, including more than 37,000 individuals from roughly 23,000 households; biomarker and genetic measures were added in 2006 [21]. Both cohorts feature rigorous quality control and long-term follow-up, providing a comparable data foundation for cross-national analyses. The CHARLS study has been approved by the Biomedical Ethics Committee of Peking University (approval number: IRB 00001052–11015), and the University of Michigan Institutional Review Board approved the HRS study. All participants provided written informed consent.

The study utilized the HRS (2012) and CHARLS (2011) as baseline. After data cleaning, participants were subsequently included or excluded as follows: Inclusion: (1) Age ≥ 60 years, (2) At least one follow-up record, (3) Key variable missing rate <10% (age, gender, CKM diagnosis: smoking, high-density lipoprotein cholesterol (HDL-C), stroke, waist circumference, heart disease, serum cystatin C, diabetes, hypertension, diabetes treatment, hypertension treatment); Exclusions: (1) Personal data missing rate >70%, (2) Death date prior to baseline survey, (3) Patients with terminal cancer, (4) Patients lacking key variables of gender or age, (5) Non-fasting baseline biochemical status in CHARLS, (6) Missing initial survey date.

### Data extraction

We extracted the following variables for each participant: (1) Demographics (age, sex, race, marital status); (2) Chronic conditions (hypertension, diabetes, heart disease, chronic kidney disease, stroke, cancer, etc.); (3) Anthropometrics and physical function (height, weight, waist circumference, grip strength, gait speed); (4) Laboratory measures (HbA1c, creatinine, HDL cholesterol, etc.); (5) Vital signs (blood pressure, heart rate); (6) Scales/scores (Frailty Index, Activities of daily living (ADL)); and (7) Survey data (baseline interview date, date and cause of death).

### Handling the missing values

Missing values were handled using multiple imputation implemented with the R package “MICE” creating 20 imputations [22, 23]. Predictive Mean Matching (PMM) was used for continuous variables, logistic regression for binary variables, and multinomial logistic regression for categorical variables with >2 levels [24]. We evaluated convergence with trace plots and assessed imputation quality by comparing densities of imputed and observed data. Estimates from the 20 datasets were combined using Rubin’s rules[25].

### Feature selection

In CHARLS, we excluded leakage-prone and non-modeling variables via a prespecified blacklist, then ran LASSO with 10-fold CV, selecting λ by the 1-SE rule across multiple α values. Predictors chosen in ≥70% of imputations were deemed stable. Variable importance was then ranked based on the absolute values of the coefficients pooled via Rubin’s rules. We assessed collinearity via Spearman correlations and Variance Inflation Factor (VIF), removing variables with |r|>0.70 based on univariable information and clinical rationale.

### Model development and validation

Using the CHARLS dataset for development, we trained six survival models: the Cox proportional hazards (COX), Competing-risks regression, Random Survival Forest (RSF), Gradient Boosting Machine (GBM Survival), Extreme Gradient Boosting (XGB Survival), and Survival Decision Tree (Survival Tree). Model were trained and tuned using 10-fold cross-validation using rigorous. Performance was assessed using the Area Under the Curve (AUC) and C-index for discrimination, and the Brier score for overall prediction error. The DeLong test was used to compare differences between models. Clinical utility was evaluated using Decision Curve Analysis (DCA). The final model was selected based on a composite criterion of high discrimination, net benefit, and low Brier score.

### External validation

We validated the best-performing prediction model identified in CHARLS within each imputed dataset of HRS, obtaining the AUC and DCA metrics for every multiply imputed (MI) dataset. We then pooled these metrics across imputations using Rubin’s rules to generate an average ROC curve and evaluated performance using the DCA curves.

### Model explanation

SHapley Additive exPlanations (SHAP) were used to interpret the ML models and quantify the impact of individual features on predictions. The SHAP feature-importance plot displays global importance, where larger mean absolute SHAP values indicate greater contribution of a feature to the model’s predictions. The SHAP summary plot visualizes each feature’s influence on the prediction across observations. By computing each feature’s contribution to the predicted outcome, SHAP provides both local and global explanations, thereby enhancing model transparency and interpretability[26].

### Statistical analyses

Continuous variables were first assessed for distribution using the Shapiro-Francia test; normally distributed variables are reported as mean ± SD, and non-normally as median (interquartile range, IQR). Categorical variables are presented as counts and percentages. All analyses were conducted in Stata/MP 18, R (Version 4.5.1), and Python (Jupyter Notebook). Statistical significance was set at P < 0.05 (two-sided).

## Results

### Baseline characteristics

**Figure 1:**
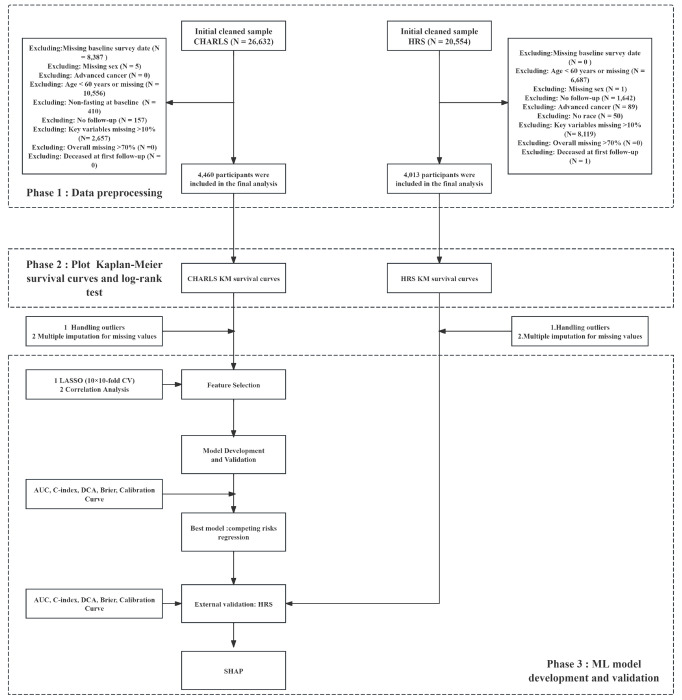
Flow Chart of the Study Design

The study included 8,473 individuals, including 4,460 from the CHARLS cohort and 4,013 from the HRS cohort (Figure 1). The mean age of the entire cohort was 69.9 ± 7.6 years (median: 69, IQR: 63-75), and 46.02% (3,899) were male.

In CHARLS (Table 1), the mean age of the cohort was 68.1 ± 6.7 years (median: 67, IQR: 63-72), and 49.26% were male. The median follow-up was 8.00 (IQR: 8.00-8.00) years, follow-up time was not normally distributed (p < 0.001), and among decedents the median survival was 5.5 (IQR: 3.5-7.3) years. There were striking differences in gender composition by CKM stage: stage 2 was predominantly female (84.6%), whereas stage 3 was predominantly male (81.5%) and had the highest prevalence of smoking (69.4%) and alcohol consumption (43.2%). With advancing CKM stage, blood pressure (systolic and diastolic), glucose, triglycerides, and waist circumference increased progressively, whereas high-density lipoprotein cholesterol (HDL-C) and glomerular filtration rate (eGFR) declined.

In HRS (Table 2), the mean age was 71.9 ± 8.0 years (median: 72, IQR: 65-77), and 57.59% were women, and Asians comprised 6.08%. Participants had a median follow-up of 8.00 (IQR: 5.83–8.00) years. CKM stages were predominantly in stages 3-4. Educational attainment was high (about 88% at high school or above). Smoking and alcohol use prevalences were 11.34% and 36.26%, respectively, though these variables had approximately 40%-46% missingness. The BMI was 28.82 kg/m^2^; waist circumference in both genders was within the range of central obesity. Median SBP/DBP was 134/80 mmHg. Comorbidities were common: hypertension (67.33%), diabetes (25.79%), heart disease (29.70%), stroke (7.63%), and chronic lung disease (11.56%). Median laboratory values were HbA1c 5.76%, HDL-C 52.3 mg/dL, Cystatin C 1.10 mg/L, and CRP 1.64 mg/L. Increasing CKM stage correlated with older age, higher adiposity and BP, greater inflammatory burden, reduced kidney function, accumulation of cardiovascular risk and clinical CVD, and poorer cognitive and functional status.

### Kaplan-Meier curves

#### CHALRS

**Figure 2:**
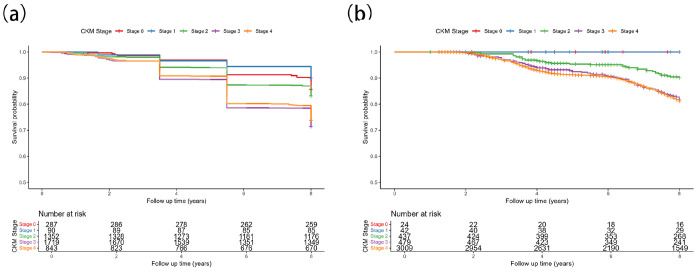
K-M Curves by Stage. (a) All-cause mortality among CHARLS over 8 years. (b) All-cause mortality among HRS over 8 years.

Figure 2 (a) presents the Kaplan–Meier curves for the first imputation 1. The log-rank test showed statistically significant separation of survival curves across CKM stages (X^2^= 83.6; df = 4; P < 0.001). In the first imputation, crude mortality by CKM stage was 14.3% (stage 0), 10.0% (stage 1), 16.8% (stage 2), 28.6% (stage 3), and 26.2% (stage 4). Pooling results from 20 imputations with Rubin’s rules yielded stage-specific 8-year mortality of 15.69% (SE = 0.0235), 9.92% (SE = 0.0341), 16.76% (SE = 0.0108), 28.51% (SE = 0.0111), and 26.19% (SE = 0.0151) for stages 0-4, consistent with the pattern observed in imputation set 1.

#### HRS

In imputation 1 (Figure 2 (b)), overall crude mortality was 14.46%. Stage-wise mortality was 0% for stages 0 and 1, 8.5% for stage 2, 14.8% for stage 3, and 15.6% for stage 4, with significant separation of survival curves by the log-rank test (X^2^ = 27.91, df = 4, P < 0.001). After pooling 20 imputations using Rubin’s rules, the 8-year mortality increased with higher CKM stage: 9.20% (SE = 0.0921) for stage 0, 4.94% (SE = 0.0406) for stage 1, 9.14% (SE = 0.0183) for stage 2, 16.57% (SE = 0.0228) for stage 3, and 18.97% (SE = 0.0083) for stage 4.

### Screening for predictive factors

**Figure 3:**
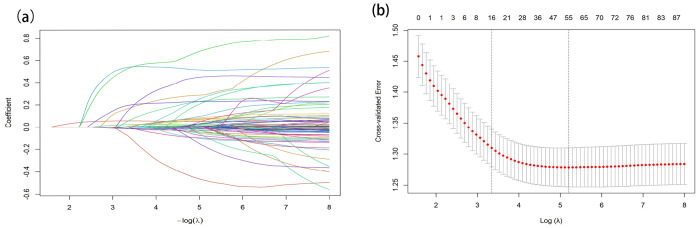
Feature selection was performed using the LASSO regression model. (a) Coefficient profile was generated based on the logarithmic (lambda) sequence, with non-zero coefficients identified at the optimal lambda. (b) The optimal lambda parameter was selected through tenfold cross-validation using the minimum criteria. The partial likelihood deviation (binomial deviation) curve relative to log(lambda) was plotted, and a vertical line was drawn at the optimal value using the 1-SE criterion.

LASSO–Cox was fitted separately in each of the 20 multiply imputed CHARLS datasets. 17 features were selected: different bathing, age, BMI, Serum Cystatin C, C-reactive protein (CRP), Mini-Mental State Examination (MMSE), resistance, sex, smoking, date, glucose, lung disease, pulse, diabetes treatment, SBP, self-rated health, and speed. The LASSO results for imputation 1 are shown in Figure 3. Extracting coefficients at the “best λ” for each imputation and pooling them using Rubin’s rules, 14 variables were significantly associated with all-cause mortality (two-sided P < 0.05): difficulty bathing, age, BMI, Cystatin C, CRP, MMSE, resistance, sex, smoking, glucose, lung disease, pulse, diabetes treatment, and SBP. Multicollinearity assessment revealed no substantive collinearity, and no variables were removed (Supplementary Figure 3). Integrating statistical significance, cross-imputation stability, and clinical interpretability, we retained 10 predictors for downstream machine-learning modeling: Cystatin C, different bathing, resistance, smoke, diabetes treatment, Speed, sex, lung disease, age, and CRP.

### Machine learning models development and performance comparison

**Figure 4:**
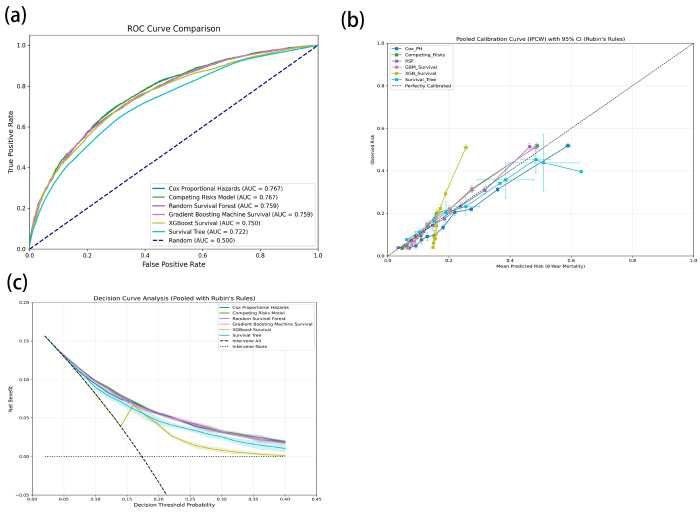
Performance of ML models predicting CKM syndrome in the development. (a) ROC curve analysis, (b) Calibration curve analysis, (c) DCA curves for each ML model.

In development ML (Table 4), the AUC analysis indicated that the Cox and competing risks regression models demonstrated the best discriminative ability, with AUC values of 76.65% (95%CI: 76.55%-76.76%) and 76.68% (95%CI: 76.57%-76.78%), respectively (Figure 4 (a)). The performance of the remaining models, in descending order of AUC, was as follows: RSF (75.86%, 95% CI: 75.73%-75.99%), GbM (75.95%, 95% CI: 75.78%-76.12%), XGBoost (74.98%, 95% CI: 74.85%-75.11%), and ST (72.22%, 95% CI: 71.29%-73.15%). In terms of predictive accuracy, the Cox model achieved the lowest Brier score (0.146), indicating the most robust performance. The DeLong test indicates that the differences between the models are not significant. The Brier scores for the competing risks model, RSF, and GbM were comparable (0.152-0.153), while XGBoost and ST exhibited greater prediction error. Calibration curve assessments revealed that the Cox and competing risks models had similar and superior calibration performance compared to the other machine learning methods (Figure 4 (b)). The DCA showed that the CRR model yielded the highest net benefit across a range of threshold probabilities (Figure 4 (c)). Consequently, the Cox model was selected for the final 8-year prediction model in this study.

### External validation

**Figure 5:**
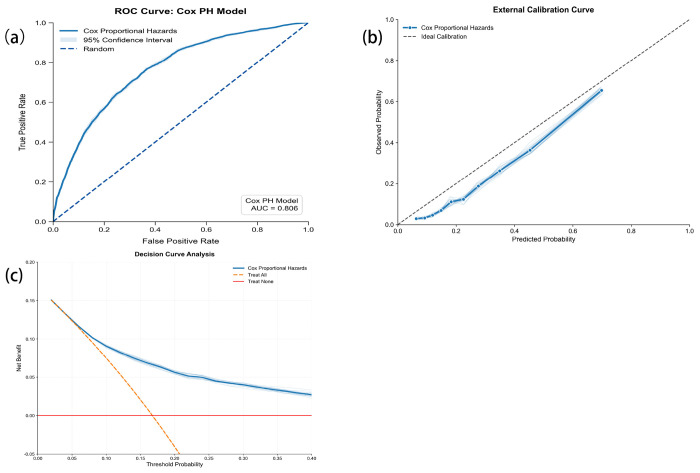
Performance of ML models predicting CKM syndrome in the external validation. (a) ROC curve analysis, (b) Calibration curve analysis, (c) DCA curves for each ML model.

In external validation using the HRS cohort (Table 5), the pooled Cox model achieved a C-index of 77.2% (95% CI: 76.8-77.6), an AUC of 76.6% (95% CI: 76.5-77.0), a Brier score of 0.125 (95% CI: 0.123-0.128), AUC (Inverse Probability of Censoring Weighting, IPCW) of 80.6% (95% CI: 80.2-81.1) , Brier score (IPCW) of 0.114 (95%CI: 0.112-0.117), which was similar to that in the internal validation (Figure 5(a)). The calibration curve closely followed the ideal line (Figure 5 (b)). DCA indicated a net clinical benefit for threshold probabilities between 1% and 40%, supporting the model’s potential clinical value (Figure 5 (c)). This successful external validation confirms the model’s generalizability and suggests it is a reliable tool for clinical application.

### Model explanation

**Figure 6:**
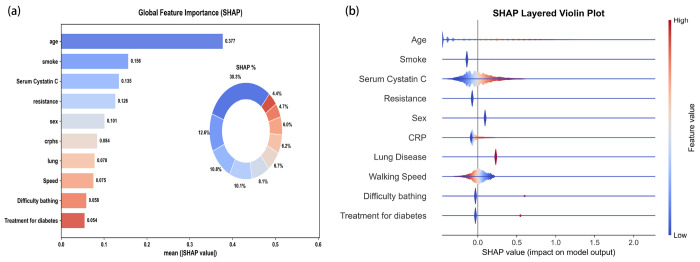
Global model explanation using SHAP method. (a) Bar and Pie plot of SHAP summary , (b) dot plot of SHAP summary. SHAP values for each feature, where blue indicates a lower feature value and red indicates a higher feature value. These plots illustrate the direction and strength of each feature’s association with the model output for 8-year all-cause mortality risk.

Figure 6 illustrates the global feature importance analysis based on SHAP values. As shown in Figure 6(a), age was the most critical predictor of 8-year all-cause mortality, possessing the highest mean absolute SHAP value (0.377) and independently contributing approximately 30.3% to the model’s explanatory power. It was followed by Smoking and Cystatin C; together, these three features accounted for over 50% of the model’s total contribution. The SHAP beeswarm plot in Figure 6(b) further reveals the directional impact of these features on risk. Age and Serum Cystatin C showed a clear positive correlation, where higher values correspond to higher SHAP values and increased mortality risk. Conversely, Walking Speed demonstrated a negative correlation, with slower speeds predicting higher mortality risk. Additionally, the presence of CRP and difficulty bathing significantly increased the risk of death.

**Figure 7:**
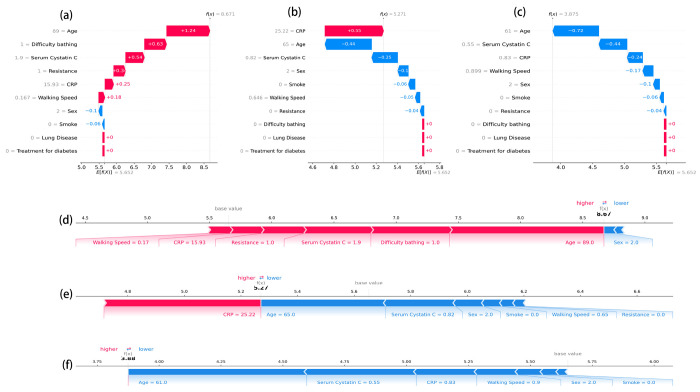
SHAP visualization of local interpretability for individual mortality risk predictions. Panels (a–c) display SHAP waterfall plots, and panels (d–f) display corresponding SHAP force plots for three representative participants selected from the study cohort. (a, d) A high-risk individual. (b, e) A medium-risk individual. (c, f) A low-risk individual. Red bars/arrows indicate features that positively contribute to the mortality risk (pushing the prediction higher), while blue bars/arrows indicate features that negatively contribute (pushing the prediction lower).

Figure 7 details individual prediction logic across three representative profiles: Low-Risk Case (Figure 7(c,f)): The waterfall plot shows that a younger age (61 years), normal Serum Cystatin C levels (0.55 mg/L), and low CRP (0.83 mg/L) acted as protective factors (blue bars), substantially reducing the risk score. Medium-Risk Case (Figure 7(b,e)): This patient had a risk score close to the average. Although highly elevated inflammation (CRP = 25.22 mg/L) significantly pushed the risk upward (red bar, +0.55), this was buffered by relatively younger age (65 years) and normal renal function (Cystatin C = 0.82 mg/L), preventing the total risk from becoming excessive. High-Risk Case (Figure 7(a,d)): This patient was classified by the model as extremely high-risk. The waterfall plot clearly visualizes the accumulation of risk factors: advanced age (89 years, +1.24), functional impairment (difficulty bathing, +0.63), impaired renal function (Cystatin C= 1.9 mg/L, +0.54), and slow walking speed (0.167 m/s). These factors combined to drive the predicted score significantly above the baseline.

### The risk calculator

We employ Jupyter Notebook for parameter invocation, with the final predictive model integrated into an Excel spreadsheet for clinical risk assessment of patients. By inputting ten current patient values, the spreadsheet automatically predicts the 8-year all-cause mortality risk for CKM patients. The Excel spreadsheet is included in the supplementary materials.

## Discussion

This study successfully developed and validated a ML prediction model for estimating 8-year all-cause mortality risk in older adults with CKM syndrome, utilizing two large, distinct cohorts from CHARLS and HRS. While we evaluated multiple advanced algorithms, the Cox proportional hazards model was selected as the final predictive tool due to its superior balance of performance, parsimony, and interpretability. The model demonstrated excellent internal and external validation performance. SHAP interpretation identified age, smoking status, cystatin C, and gait speed as key risk drivers, underscoring the model’s clinical utility and robustness across diverse populations.

The cohorts exhibited notable demographic and clinical differences: HRS population was older, had a higher level of education, and exhibited a greater burden of chronic conditions, the CHARLS was somewhat younger but had a higher proportion of smokers and lower educational attainment. The distribution of CKM stages further illuminated population-specific patterns. The concentration in stages 2–4 within HRS, and the gender difference, mirrors findings from US-based studies [8, 9]. The predominance of stage 3 in the elderly CHARLS cohort, while differing from some broader summaries [10, 27], is corroborated by data focused on older adults and aligns with Chen et al.’s report of advanced staging in this age group [11].

Despite these disparities, both cohorts demonstrated a consistent graded trend: higher CKM stages were associated with greater mortality risk, supporting the generalizability of the AHA-proposed CKM staging system across diverse populations [5]. It is noteworthy that although the median age in the CHARLS was lower than in HRS, the overall mortality rate was higher. This discrepancy may be partly explained by the lower education levels and higher smoking rates within the CHARLS cohort [28, 29]. Consequently, these findings underscore a potential need for healthcare providers in China to prioritize the earlier identification and intervention of high-risk CKM populations compared to practices in the United States.

Feature selection plays a critical role in the development of machine learning model [30]. Our comparative analysis, leveraging 20 imputations and 10-fold cross-validation, revealed that the Cox and competing risks regression models offered marginally better discrimination than several machine learning alternatives. The Cox model was prioritized as the final tool, because it achieved the best performance, such as C-index, AUC and Brier Score and demonstrated the highest net benefit across a broad range of threshold probabilities (1%–40%) in DCA. External validation in the HRS cohort further affirmed the model’s robustness and transferability. These findings suggest that in real-world clinical scenarios—often characterized by limited variables and competing risks—a structurally simpler, interpretable model like the Cox regression is often superior to “black-box” algorithms for long-term survival prediction.

SHAP analysis provided a quantitative assessment of both feature importance and the direction of their effects. In this study, age emerged as the strongest and most consistent non-modifiable risk factor, aligning with its role in the majority of chronic conditions [31]. In this study, smoking refers to having a history of smoking and being a current smoker. Smoking, a major modifiable risk factor, demonstrated a significant impact, which is consistent with its well-established, profound effects on cardiovascular, renal, and metabolic diseases, and its role in exacerbating conditions like hypertension and diabetes that further impair renal function [32, 33].

Cystatin C ranked as a top predictor of intervention in our model. Current study con^rms that elevated Cystatin C is significantly associated with an increased risk of cardiovascular diseases, even after adjusting for traditional risk factors [34, 35]. It is implicated in vascular wall remodeling, inflammation, and atherosclerosis by inhibiting cysteine proteases, leading to extracellular matrix degradation, plaque instability, and thrombosis [35–37]. Furthermore, Cystatin C is recognized as an independent risk factor for CKD progression and a predictor of renal function decline and mortality [38], and it has significant associations with metabolic diseases [39], solidifying its role as a central biomarker in the CKM spectrum. SHAP importance scores directly demonstrate the importance of Cystatin C in the prediction. The force plots visually demonstrate how protective factors (e.g., younger age, good functional status) can offset high-risk features (e.g., smoke, elevated Cystatin C) in individual patients. This makes SHAP a valuable tool for visualizing risk and facilitating shared decision-making in clinical practice.

Gait speed represents a modifiable functional indicator. Walking has been demonstrated to reduce the risk or severity of a range of health outcomes, including all-cause mortality, cardiovascular and cerebrovascular diseases and type 2 diabetes [40–44]. The relationship with mortality is non-linear, even a modest increase (e.g., from 0.6 to 0.7 m/s) can reduce risk by 10% [45]. Consequently, our results strongly support the implementation of targeted physical function exercises for patients. We posit that interventions designed to improve gait speed can initiate a positive feedback loop, whereby enhanced mobility leads to broader functional gains, thereby creating a virtuous cycle that mitigates the 8-year all-cause mortality risk [46].

The stable net benefit of our model, as demonstrated by DCA across a wide threshold range (1%-40%), validates its application for risk stratification in clinical practice. This supports a stratified intervention approach: younger seniors with risk driven by Cystatin C and smoking require aggressive management of these modifiable factors, whereas those with functional decline, indicated by slow gait speed, should be referred for prompt physical therapy and rehabilitation. Crucially, successful implementation demands that these strategies be tailored to local demographics and risk factor profiles—such as educational attainment and smoking rates—to ensure they are contextually relevant and thus more likely to succeed.

This work provides a novel, dually-validated, and interpretable prediction model for 8-year mortality in CKM, derived from major international cohorts. The research strengths include a focus on readily obtainable variables, methodological rigor through multiple imputation, the appropriate application of a competing risks model that showed strong transportability, and the integration of SHAP values for individual-level interpretation.

While this study has limitations, the absence of data on Peripheral Artery Disease (PAD) and key cardiac biomarkers represents an important omission. Direct comparison between cohorts is complicated by the use of different assays for estimating renal function (creatinine in CHARLS vs. cystatin C in HRS). Estimates for CKM stages 0–1 were unstable due to their low prevalence and few events in HRS. The historical nature of the data (2011–2012) may limit contemporary relevance, and the observational design cannot preclude residual confounding. Prospective studies are needed to confirm the model’s applicability across diverse age ranges, healthcare settings, and ethnicities. Future studies should also investigate interventions tailored to the specific characteristics of different populations.

Future research should include multi-center, prospective studies to validate the model’s clinical utility. Integrating a broader panel of cardiorenal biomarkers and vascular phenotypes (e.g., PAD), alongside multi-modal data from imaging and wearable devices, could improve early-stage detection and risk stratification. Finally, in-depth investigations into interventions tailored to specific population groups are critically needed.

## Conclusion

This study developed and validated the Cox model that robustly identifies the graded mortality risk associated with CKM staging across international cohorts, confirmed by its discrimination, calibration, and clinical utility. The model identifies age, smoking, and cystatin C as pivotal risk drivers, and gait speed and functional status as critical indicators. Ultimately, this study provides a practical, easy-to-use tool for personalized risk assessment, facilitating more precise clinical decision-making for CKM management in aging populations globally.

## Supplementary Material

This is a list of supplementary files associated with this preprint. Click to download.

SupplementaryMaterial.docx

8YearMortalityCalculator.xlsx

## Figures and Tables

**Table 1:** Baseline Characteristics of CHARLS 2011 Participants Across CKM Stages

Characteristic	Overall (n=4460)	Stage 0(n=154)	Stage 1(n=240)	Stage 2 (n=1145)	Stage 3 (n=1439)	Stage 4(n=663)	Missing (%)
**Demographic Characteristics**							
**Age (year)**	67 (63,73)	64 (62, 68)	65 (62, 70)	68 (64, 74)	67 (63, 72)	67 (63, 72)	0
**Gender**							0
**Man**	2197 (49.26%)	94 (61.04)	107 (44.58)	176 (15.37)	1,173 (81.51)	300 (45.25)	
**Woman**	2263 (50.74)	60 (38.96)	133 (55.42)	969 (84.63)	266 (18.49)	363 (54.75)	
**Marital status, n (%)**							0
**Single**	41 (0.92)	2 (1.30)	1 (0.42)	3 (0.26)	23 (1.60)	1 (0.15)	
**Married**	3533 (79.22)	135 (87.66)	201 (83.75)	880 (76.86)	1,174 (81.58)	509 (76.77)	
**Divorced**	51 (1.14)	2 (1.30)	5 (2.08)	7 (0.61)	23 (1.60)	7 (1.06)	
**Widowed**	835 (18.72)	15 (9.74)	33 (13.75)	255 (22.27)	219 (15.22)	146 (22.02)	
**Education, n (%)**							5 (0.11)
**Illiterate**	1708 (38.30)	49 (31.82)	95 (39.58)	541 (47.25)	425 (29.53)	217 (32.73)	
**Incomplete primary education or No formal education**	917 (20.56)	36 (23.38)	58 (24.17)	219 (19.13)	333 (23.14)	131 (19.76)	
**Completed primary education**	1078 (24.17)	38 (24.68)	50 (20.83)	240 (20.96)	417 (28.98)	185 (27.90)	
**Completed junior secondary education**	507 (11.37)	23 (14.94)	29 (12.08)	101 (8.82)	184 (12.79)	87 (13.12)	
**Senior secondary education**	180 (4.04)	5 (3.25)	4 (1.67)	34 (2.97)	63 (4.38)	27 (4.07)	
**College or Above**	65 (1.46)	2 (1.30)	4 (1.67)	10 (0.87)	16 (1.11)	16 (2.41)	
**Lifestyle Factors**							
**Smoke, n (%)**	1858 (41.66)	59 (38.31)	78 (32.50)	157 (13.71)	999 (69.42)	272 (41.03)	0
**Drinking, n (%)**	1338 (69.98)	56 (36.36)	79 (32.92)	224 (19.56)	622 (43.22)	145 (21.87)	1 (0.02)
**Anthropometrics & Vital Signs**							
BMI (kg/m^2^)	22.60 (20.29, 25.29)	19.77 ± 1.75	21.87 ± 2.76	23.00 (20.73, 25.60)	22.56 (20.18, 25.17)	23.54 (20.91, 26.60)	631 (13.71)
**Waist circumference in men, cm**	83.2(77.0,91.0)	76.83 ± 5.39	81.83 ± 7.74	83.41 ± 8.63	84.00 (77.80, 91.40)	86.40 (78.00, 95.60)	229 (10.42)
**Waist circumference in women, cm**	86.0(78.2,93.0)	74.40 (70.20, 76.00)	82.79 ± 8.32	86.25 (79.00, 93.00)	90.44 ± 9.45	87.92 ± 10.63	280 (12.37)
**SBP, mm Hg**	138 (123, 154)	112.85 ± 8.50	118.00 (112.00, 123.00)	138.00 (126.00, 150.00)	145.00 (131.00, 163.00)	140.00 (124.00, 156.00)	490 (10.99)
**DBP, mm Hg**	77 (69. 85)	66.38 ± 6.85)	69.00 (63.00, 73.00)	78.00 (70.00, 85.00)	80.00 (73.00, 88.00)	78.00 (70.00, 86.00)	475 (10.65)
**Pulse, beats/min**	74 (67, 81)	72.96 ± 10.18	73.00 (68.00, 80.00)	74.00 (68.00, 80.50)	73.00 (67.00, 81.00)	74.00 (67.00, 82.00)	493 (11.05)
**Diseases**							
**Hypertension, n (%)**	1468 (32.91)	0	0	291 (25.41)	534 (37.11)	358 (54.00)	11 (0.25)
**Diabetes mellitus, n (%)**	308 (6.91)	0	0	40 (3.49)	116 (8.06)	91 (13.73)	17 (0.38)
**Heart disease, n (%)**	727 (16.3)	0	0	0	0	581 (87.63)	1 (0.02)
**Stroke, n (%)**	156 (3.5)	0	0	0	0	106 (15.99)	0
**Laboratory Findings**							
**Glucose (mg/dl)**	102.78(95.22,114.48)	92.16 (88.56, 96.48)	104.22 (97.02, 109.80)	103.50 (96.12, 114.30)	103.68 (95.94, 117.72)	103.86 (96.12, 116.28)	4 (0.09)
**HbA1c (%)**	5.2(4.9,5.5)	5.00 (4.80, 5.30)	5.21 ± 0.40	5.20 (4.90, 5.50)	5.20 (4.90, 5.50)	5.20 (4.90, 5.50)	22 (0.49)
**Total cholesterol (mg/dl)**	192.33(168.17,218.43)	180.54 (160.44, 204.12)	191.75 (168.56, 215.72)	197.94 (173.97, 223.45)	190.21 (167.01, 214.95)	192.53 (168.94, 218.04)	0
**LDLC cholesterol (mg/dl)**	117.14(95.49,139.95)	107.47 (88.14, 128.74)	115.98 (97.23, 135.50)	121.01 (98.20, 144.40)	115.21 (94.33, 138.40)	117.14 (95.49, 140.34)	8 (0.18)
**HDLC cholesterol (mg/dl)**	50.26(40.98,60.70)	59.92 (52.58, 68.81)	61.08 (53.16, 69.97)	52.19 (43.69, 61.47)	46.39 (38.66, 57.22)	48.71 (38.27, 59.15)	35 (0.78)
**Triglycerides (mg/dl)**	103.55(74.34,146.91)	75.23 (58.41, 98.24)	77.00 (62.84, 93.81)	114.17 (79.65, 159.30)	103.55 (74.34, 153.99)	112.40 (80.54, 155.76)	1 (0.02)
**Creatinine (mg/dl)**	0.780(0.667,0.915)	0.78 ± 0.16	0.75 ± 0.15	0.71 (0.63, 0.81)	0.86 (0.75, 0.98)	0.79 (0.68, 0.95)	1 (0.02)
**Serum Cystatin C (mg/l)**	1.07(0.94,1.23)	1.08 ± 0.19	1.02 ± 0.17	1.02 (0.90, 1.16)	1.12 (0.96, 1.29)	1.09 (0.96, 1.26)	711 (15.94)
**eGFR (mL/min/1.73 m^2^)**	95.88(81.31,112.52)	101.37 (89.32, 116.71)	102.16 (88.20, 120.16)	98.96 (84.50, 115.05)	92.76 (78.85, 109.17)	92.59 (79.31, 108.31)	1 (0.02)
**CKM Staging Components**							
**High predicted 10-y CVD risk**							544 (12.22)
**FRS < 10%**	837 (18.77)	81 (52.60)	155 (32.99)	384 (33.54)	0	120 (18.10)	
**10 < FRS < 20**	1373 (30.78)	73 (47.40)	85 (67.01)	761 (66.46)	1 (0.07)	198 (29.86)	
**FRS ≥ 20**	2250 (50.45)	0	0	0	1,438 (99.93)	345 (52.04)	
**Clinical CVD**	842 (18.88)	0	0	0	0	663 (100)	544 (12.22)
**MMSE**	12 (7,16)	11.85±6.00	11.57±5.92	11 (7,16)	13 (8,17)	12 (7,17)	0
Difficultly Bath	20 (4.64)	3 (1.95)	4 (4.67)	35 (3.06)	33 (2.29)	44 (6.64)	0
**Resistance**	1,021 (22.89)	19 (12.34)	38 (15.83)	225 (16.65)	268 (18.62)	224 (33.79)	35 (0.78)
**Walking speed**	4.3 (3.51, 5.5)	4.05 (3.4, 4.9)	4.06(3.46, 5.13)	4.25 (3.5,5.5)	4.25 (3.41, 5.4)	4.52 (3.72, 5.81)	695 (15.58)

BMI: Body Mass Index; DBP: diastolic blood pressure; SBP: Systolic blood pressure; HbA1c: Glycated Hemoglobin; LDLC cholesterol: Low-Density Lipoprotein Cholesterol; HDLC cholesterol: High-Density Lipoprotein Cholesterol; eGFR: glomerular filtration rate; FRS: Framingham Risk Score

**Table 2:** Baseline Characteristics of HRS 2012 Participants Across CKM Stages

Characteristic	Overall (n=4,013)	Stage 0 (n=0)	Stage 1(n=111)	Stage 2(n=1667)	Stage 3(n=804)	Stage 4(n=1342)	Missing (%)
**Demographic Characteristics**							
**Age (year)**	72 (65,77)	-	65 (62, 72)	70 (64, 76)	72 (65, 77)	74 (68, 79)	0
**Gender**							0
**Man**	1,702 (41.41)	-	40 (36.04)	393 (23.58)	594 (73.97)	645 (48.06)	
**Woman**	2,311 (57.59)	-	71 (63.96)	1,271 (76.38)	209 (26.03)	697 (51.94)	
**Race**							0
**Asian**	244 (6.08)	-	8 (7.21)	113 (6.79)	58 (7.22)	60 (4.47)	
**Non-Asian**	3769 (93.92)	-	103 (92.79)	1,551 (93.21)	745 (92.78)	1,282 (95.53)	
**Marital status, n (%)**							4 (0.1)
**Single**	139 (3.46)	-	2 (1.80)	66 (3.97)	27 (3.36)	38 (2.83)	
**Married**	2, 360 (58.81)	-	81 (72.97)	942 (56.61)	502 (62.52)	779 (58.05)	
**Divorced**	592 (14.75)	-	15 (13.51)	246 (14.78)	118 (14.69)	192 (14.31)	
**Widowed**	918 (22.88)	-	13 (11.71)	410 (24.64)	154 (19.18)	331 (24.66)	
**Education, n (%)**							0
**Illiterate**	29 (0.72)	-	0	17 (1.02)	3 (0.37)	9 (0.67)	
**Incomplete primary education or No formal education**	101 (2.52)	-	3 (2.7)	42 (2.52)	26 (3.24)	30 (2.24)	
**Completed primary education**	66 (1.64)	-	2 (1.8)	21 (1.26)	21 (2.62)	22 (1.64)	
**Completed junior secondary education**	293 (7.30)	-	3 (2.7)	100 (6.01)	73 (9.09)	113 (8.42)	
**Senior secondary education**	1,651 (41.14)	-	33 (29.73)	629 (37.80)	344 (42.84)	607 (45.23)	
**College or Above**	1,873 (46.67)	-	70 (63.06)	855 (51.38)	336 (41.84)	561 (41.80)	
**Lifestyle Factors**							
**Smoke, n (%)**	455 (11.34)	-	10 (9.01)	99 (5.95)	193 (24.03)	143 (10.66)	1,589 (39.60)
**Drinking, n (%)**	1,455 (36.26)	-	52 (46.85)	595 (35.76)	340 (42.34)	426 (31.74)	1,862 (46.40)
**Anthropometrics & Vital Signs**							
**BMI (kg/m^2^)**	28.82 (25.43, 32.98)	-	26.60 (25.02, 29.93)	28.72 (25.22, 32.97)	28.97 (25.92, 32.86)	29.41 (25.87, 33.61)	89 (2.22)
**Waist circumference in men, cm**	104.14 (96.52, 113.03)	-	100.89 ± 12.71	101.6 (95.25, 110.49)	105.41 (96.52, 113.03)	106.68 (97.79, 114.94)	229 (10.42)
**Waist circumference in women, cm**	97.79 (88.9, 107.95)	-	90.98 ± 10.67	97.79 (88.9, 107.32)	101.6 (91.44, 111.76)	99.06 (90.17, 110.49)	137 (3.41)
**SBP, mmHg**	134 (122, 148)	-	115 (110, 123)	133 (122, 146)	142 (131, 156)	134 (122, 148)	137 (3.41))
**DBP, mmHg**	80 (73, 88)	-	72 (67, 75)	81 (74.5, 89)	83.50 ± 11.34	78 (70, 87)	115 (2.87)
**Pulse, beats/min**	69 (62, 77)	-	69 (64, 76)	70 (63, 78)	71 (63, 78)	67.5 (60, 76)	120 (2.99)
**Diseases**							
**Hypertension, n (%)**	2, 702 (67.33)	-	0	1,060 (63.70)	594 (73.97)	1,048 (78.09)	5 (0.12)
**Diabetes mellitus, n (%)**	1, 035 (25.79)	-	0	306 (18.39)	278 (34.62)	451 (33.61)	3 (0.07)
**Heart disease, n (%)**	1, 192 (29.70)	-	0	0	1 (0.12)	1,190 (88.67)	2 (0.05)
**Stroke, n (%)**	306 (7.63)	-	0	0	0	306 (22.80)	0
**Lung disease, n (%)**	464 (11.56)	-	5 (4.50)	116 (6.91)	98 (12.20)	239 (17.81)	2 (0.05)
**Laboratory Findings**							
**HbA1c (%)**	5.76 (5.3, 6.21)	-	5.51 ± 0.44	5.6 (5.3, 6.06)	5.76 (5.45, 6.52)	5.76 (5.45, 6.37)	68 (1.69)
**Total cholesterol (mg/dl)**	189.94 (165.38, 218.37)	-	214.86±34.43	198.34 (173.13, 226.13)	187.35 (160.85, 215.79)	179.6 (156.98, 208.03)	0
**HDLC cholesterol (mg/dl)**	52.3 (42.31, 63.01)	-	60.86 (53.01, 67.29)	55.87 (46.59, 65.86)	48.02 (38.74, 59.44)	49.44 (40.16, 59.44)	24 (0.60)
**Serum Cystatin C (mg/l)**	1.1 (0.9, 1.33)	-	0.96 (0.85, 1.06)	1.08 (0.88, 1.3)	1.08 (0.88, 1.33)	1.16 (0.98, 1.41)	287 (7.15)
**eGFR (mL/min/1.73 m^2^)**	63.17 (48.42, 82.08)	-	76.21 (66.89, 90.24)	64.15 (49.33, 83.35)	65.29 (49.15, 86.70)	58.25 (44.54, 74.40)	287 (7.15)
**Crp**	1.64 (0.83, 3.22)	-	1.16 (0.65, 2.02)	1.63 (0.84, 3.15)	1.66 (0.85, 3.33)	1.78 (0.87, 3.37)	314 (7.82)
**CKM Staging Components**							
**High predicted 10-y CVD risk**							1,745 (43.48)
**FRS < 10%**	344 (8.56)	-	38 (34.23)	183 (11.00)	4 (0.5)	84 (6.26)	
**10 < FRS < 20**	595 (14.81)	-	19 (17.12)	379 (22.78)	2 (0.25)	178 (13.26)	
**FRS ≥ 20**	1,333 (33.18)	-	0	0	781 (97.26)	551 (41.06)	
**Clinical CVD**	1,344 (33.49)	-	0	0	1 (0.12)	1342 (100)	2,669 (66.51)
**MMSE**	18 (15, 21)	-	20.14±4.30	19 (16, 22)	18 (15, 21)	18 (15, 21)	1,539 (38.35)
**Difficultly Bath**	207 (5.16)	-	0	57 (3.43)	33 (4.11)	115 (8.57)	1905 (47.47)
**Resistance**	762 (18.99)	-	7 (6.31)	248 (14.90)	128 (15.94)	371 (27.65)	3 (0.07)
**Speed**	3.37 (2.87, 4.24)	-	2.91 (2.46, 3.4)	3.25 (2.75, 4.11)	3.22 (2.87, 3.97)	3.64 (2.93, 4.55)	1240 (30.90)

BMI: Body Mass Index; DBP: diastolic blood pressure; SBP: Systolic blood pressure; HbA1c: Glycated Hemoglobin; HDLC cholesterol: High-Density Lipoprotein Cholesterol; eGFR: glomerular filtration rate; FRS: Framingham Risk Score

**Table 3:** Association between CKM Stages and 8-Year All-Cause Mortality

Variable	Participants	Deaths	Pooled 8-Year Mortality Rate (SE), %
**CHARLS**			
CKM stage 0	281	47	16.5% (0.023)
CKM stage 1	94	10	10.8% (0.033)
CKM stage 2	1,352	224	17.7% (0.011)
CKM stage 3	1,716	503	30.3% (0.011)
CKM stage 4	845	226	28.2% (0.016)
**HRS**			
CKM stage 0	24	0	9.2% (0.092)
CKM stage 1	42	0	4.9% (0.041)
CKM stage 2	437	37	9.1% (0.018)
CKM stage 3	479	71	16.6% (0.023)
CKM stage 4	3,009	469	19.0% (0.008)

**Table 4:** Evaluation of model performance in the development

Model Type	Algorithm	C-index	AUC	Brier Score	AUC (IPCW)	Brier Score (IPCW)
Linear Models						
Penalized Cox	Competing Risks Model	0.748 (0.747-0.749)	0.767 (0.766-0.768)	0.152 (0.151-0.152)	0.759 (0.759–0.760)	0.122 (0.122–0.123)
Standard Cox	Cox proportional hazards model	0.748 (0.747-0.748)	0.767 (0.765-0.768)	0.146 (0.146-0.147)	0.759 (0.759–0.760)	0.124 (0.124–0.124)
Ensemble ML						
Gradient Boosting	Gradient Boosting Machine for survival	0.741 (0.739-0.743)	0.759 (0.758-0.761)	0.153 (0.153-0.154)	0.753 (0.750–0.755)	0.124 (0.124–0.125)
Random Forest	Random Survival Forest	0.740 (0.739-0.742)	0.759 (0.757-0.760)	0.152 (0.151-0.152)	0.755 (0.752–0.757)	0.124 (0.124–0.124)
XGBoost	Extreme Gradient Boosting for survival	0.732 (0.731-0.734)	0.750 (0.748-0.751)	0.170 (0.169-0.170)	0.745 (0.742–0.748)	0.136 (0.135–0.136)
Tree Based						
Survival Tree	Survival Decision Tree	0.707 (0.699-0.715)	0.722 (0.713-0.731)	0.157 (0.156-0.159)	0.719 (0.709–0.729)	0.130 (0.128–0.131)

AUC: Area Under the Curve; AUC (IPCW): Area Under the Curve with Inverse Probability of Censoring Weighting; Brier Score (IPCW): Brier Score with Inverse Probability of Censoring Weighting

**Table 5:** Evaluation of the model performances in the external validation set

Metric	Mean	95% CI
C-index	0.772	0.768-0.776
AUC	0.766	0.762–0.770
AUC (IPCW)	0.806	0.802–0.811
Brier Score	0.125	0.123–0.128
Brier Score (IPCW)	0.114	0.112–0.117

AUC: Area Under the Curve; AUC (IPCW): Area Under the Curve with Inverse Probability of Censoring Weighting; Brier Score (IPCW): Brier Score with Inverse Probability of Censoring Weighting

## Data Availability

The raw data and questionnaires for this study are available online: CHARLS (https://charls.pku.edu.cn/), HRS (https://hrs.isr.umich.edu/data-products).

## Abbreviations

ADL: Activities of daily living
AHA: American Heart Association
AI: Artificial Intelligence
AUC: Area Under the Receiver Operating Characteristic Curve
BMI: Body Mass Index
BP: Blood Pressure
CHARLS: China Health and Retirement Longitudinal Study
CI: Confidence Interval
CKD: Chronic Kidney Disease
CKM: Cardiovascular-Kidney-Metabolic Syndrome
Cox model: Cox Proportional Hazards Model
CRP: C-reactive Protein
CV: Cross-Validation
CVD: Cardiovascular Disease
DBP: Diastolic Blood Pressure
DCA: Decision Curve Analysis
eGFR: Estimated Glomerular Filtration Rate
EPV: Events Per Variable
GBD: Global Burden of Disease
GbM: Gradient Boosting Machine
HbA1c: Glycated Hemoglobin
HDL-C: High-Density Lipoprotein Cholesterol
HR: Hazard Ratio
HRS: Health and Retirement Study
IPCW: Inverse Probability of Censoring Weighting
IQR: Interquartile Range
KM: Kaplan–Meier
LASSO: Least Absolute Shrinkage and Selection Operator
MI: Multiple Imputation
ML: Machine Learning
MMSE: Mini-Mental State Examination
NCDs: Noncommunicable Diseases
NHANES: National Health and Nutrition Examination Survey
PAD: Peripheral Artery Disease
ROC: Receiver Operating Characteristic
RSF: Random Survival Forest
SBP: Systolic Blood Pressure
SD: Standard Deviation
SE: Standard Error
SHAP: SHapley Additive exPlanations
ST: Survival Decision Tree
TRIPOD: Transparent Reporting of a multivariable prediction model for Individual Prognosis or Diagnosis
UK: United Kingdom
US: United States
VIF: Variance Inflation Factor
XGB: Extreme Gradient Boosting
